# Study on Mechanical Properties of Polyurethane Cross-Linked P(E-co-T)/PEG Blended Polyether Elastomer

**DOI:** 10.3390/polym14245419

**Published:** 2022-12-11

**Authors:** Puyu Jin, Aimin Pang, Rongjie Yang, Xiaoyan Guo, Jiyu He, Jinxian Zhai

**Affiliations:** 1 School of Materials Science and Engineering, Beijing Institute of Technology, Beijing 100081, China; 2 Key Laboratory of Aerospace Chemical Power Technology, Xiangyang 441003, China

**Keywords:** polyurethane cross-linked elastomer, polyether blend, crystallinity, mechanical properties

## Abstract

To improve the mechanical properties of polyurethane cross-linked poly (ethylene oxide-co-tetrahydrofuran) (P(E-co-T)) elastomers at room temperature, using poly (ethylene oxide-co-tetrahydrofuran) and high-molecular-weight polyethylene glycol (PEG) as raw materials and polyisocyanate N100 as curing agent, a series of polyurethane cross-linked blended polyether elastomers were prepared by changing the elastomer-curing parameter R value (n(-NCO)/n(-OH)) and P(E-co-T)/PEG ratio. Equilibrium swelling measurements showed that the chemical cross-linkage of the elastomers tended to decrease with the decreasing R value, the average molecular weight (Mc) of the network chain increased, and the density of the network chain (N0) decreased. Wide-angle X-ray diffraction (WAXD) and differential scanning calorimetry (DSC) tests showed that PEG chain segments within the elastomers crystallized at room temperature, while the crystallinity increased with decreasing R value and increasing PEG content. The mechanical property tests showed that the elongation at break tended to decrease with increasing R value; the tensile strength first increased and then decreased. At R value 0.9, the elastomer presented good comprehensive mechanical properties. In addition, the mechanical properties of polyurethane cross-linked P(E-co-T)/PEG blended polyether elastomer showed an increasing trend with the increase in PEG content when the curing parameter of 0.9 remained unchanged.

## 1. Introduction

Polyurethane elastomer is a kind of polymer material made from polyisocyanate and macromolecular polyol, which has good chemical and mechanical properties and is widely used in many fields, such as the automobile industry, the printing industry, the leather industry, and so on [1,2,3]. Among them, thermoset polyurethane elastomer is a very important category of polyurethane elastomers, which has the advantages of good topological structure stability, chemical resistance, wear resistance, and thermal stability [3,4,5], and can be applied in the aerospace industry and other industries [6,7,8].

Until now, the mechanical properties of thermoset polyurethane elastomers have been focused on the chemical crosslinking density of the elastomer [5,9,10,11], the chemical structure of the cross-linking point [5,12,13], and the type of isocyanate curing agent used [14,15,16]. Meiorin et al. [5] have used MDI (4,4′-diphenylmethane diisocyanate) as the curing agent, and glycerol and trimethylolpropane as the cross-linking agents, and prepared a series of polyurethane elastomers with different crosslinking density, mechanical properties, and thermal stability. Zhai et al. [12] have prepared a series of polyurethane crosslinked poly(3,3-bis(azidomethy)oxetane-tetrahydrofuran) elastomers with identical chemical crosslinking networks and different chemical structures at the crosslinking points, and the results show that the hydrogen bonding between the crosslinking points has an important effect on the mechanical properties of the elastomers. Liu et al. [14] have used isophorone diisocyanate and dicyclohexylmethane 4,4′-diisocyanate as curing agents to prepare thermoset polyurethane elastomers, and the results show that the structure of the isocyanate curing agents has an important influence on the mechanical properties of the elastomers. Kojio et al. [15] have used TEGDI (1,2-diisocyanatoethoxyethane) and HDI (1,6-diisocyanatohexane) as curing agents, respectively, and prepared polyurethane elastomers, and the results show that the elastomers prepared by TEGDI have lower tensile modulus and higher elongation than HDI due to the flexible ether bond structure in the TEGDI molecule. Additionally, recent studies have shown that polyurethane crosslinking reaction kinetics is also an important factor affecting the chemical crosslinking network and the macroscopic properties of elastomers [17,18].

In addition to using the chemical crosslinking structures to modulate the mechanical properties of polyurethane elastomers, using the crystallization behavior of macromolecular soft segments is also an important method for preparing elastomers with excellent mechanical properties [19,20,21]. Eceiza et al. [22] have reported that the strain-induced crystallization of polycarbonate chain segments can strengthen the physical crosslinking interaction between the chains and improve the mechanical properties of the elastomer. Anokhin et al. [23] blended PCL (poly-ε-caprolactone) and PBA (poly(1,4-butylene adipate)), whose crystallization behavior is different from PCL, and obtained a series of polyurethane chain-extended PCL/PBA elastomers with different mechanical properties. Given that the crystallinity is related to elastomer thermal history, Gorbunova et al. [24] subjected polyurethane chain-extended PBA elastomers to different heat treatments to modulate the crystallinity of PBA segments within the elastomers and achieved elastomers with different mechanical properties. However, due to the different thermodynamic properties of macromolecule chains, the blended parts within thermoplastic elastomers would show some de-mixing phenomena during long-term storage because of molecular chain slow creep, causing the mechanical properties to change [25,26,27]. In contrast, within a thermoset elastomer, the molecular chains are fixed into a crosslinked network structure by a crosslinking agent, and even if the resultant material tends toward de-mixing thermodynamically, the de-mixing phenomena would not occur. In combination with the soft segment crystallization mechanism of thermoplastic elastomers and the thermal stability advantage of thermoset elastomers, the mechanical properties of thermoset elastomers could be further improved.

**Figure 1:**
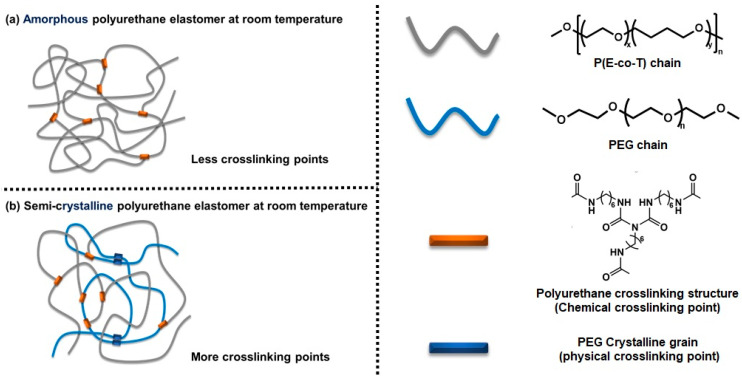
Aggregations of polyurethane crosslinked thermoset elastomers.

Poly(ethylene oxide-*co*-tetrahydrofuran) is amorphous at room temperature, and it is difficult to form effective crystallization physical crosslinking [28]; large molecule polyethylene glycol has a regular molecular chain structure, is prone to crystallizing, and forms physical crosslinks [29]. Given that P(E-co-T) and PEG have similar chemical composition of chain segments and similar solubility parameters, they have good mutual solubility [30]. In this paper, polyurethane cross-linked elastomers were prepared by blending P(E-co-T) and PEG and the influence mechanism of the elastomer crystallization behaviors on the mechanical properties was investigated in detail (Figure 1).

## 2. Materials and Methods

### 2.1. Materials

Hydroxyl terminated poly (ethylene oxide-co-tetrahydrofuran) (P(E-co-T), Mn 4038 g mol^−1^, hydroxyl value 0.467 mmol g^−1^) and polyisocyanate cross-linker N100 (Mn 741 g mol^−1^, isocyanate concentration 5.36 mmol g^−1^) were provided by Luoyang Liming Chemical Research Institute. Polyethylene glycol (PEG, Mn 4000 g mol^−1^, hydroxyl value 0.5 mmol g^−1^,) was purchased from Aladdin Chemical Company. Curing catalyst ditin butyl dilaurate (T12) was purchased from Maclin Chemical Company. 

### 2.2. Preparation of Elastomers

Fixing PEG content at 12%, the formulations of blended polyether elastomers with different curing parameter R values (the molar ratios of the isocyanate group to the hydroxyl group, n(-NCO)/n(-OH)) are listed in Table 1. According to Table 1, all components were uniformly mixed at 65 °C, poured into Teflon molds, and degassed in a vacuum. The resultant mixtures were cured at 65 °C until the isocyanate absorption peak at 2260 cm^−1^ disappeared by FTIR analysis. Thus, a series of polyurethane cross-linked blended polyether elastomers S1-1~S1-5 were obtained.

Fixing R value at 0.9, the elastomer formulations with different PEG contents are listed in Table 2. Similarly, as shown in Table 2, all components were uniformly mixed at 65 °C, poured into Teflon molds, and degassed in a vacuum. The mixtures were cured at 65 °C until the isocyanate absorption peak at 2260 cm^−1^ disappeared by FTIR analysis. Thus, a series of polyurethane cross-linked blended polyether elastomers S2-1~S2-4 were obtained.

### 2.3. Characterization

#### 2.3.1. FTIR

Elastomer samples were tested at 20 °C by using a Nicolet 6700 infrared spectrometer (Thermo) with an attenuated total reflection (ATR) assembly for FTIR investigation. The tests were carried out with a resolution of 2 cm^−1^, 32 scans, and a scan range of 4000–500 cm^−1^.

#### 2.3.2. Density Test

The densities of elastomer samples were tested by AccuPyc II 1345 true density analyzer (Micromeritics). Nitrogen was used as a test gas, and the test temperature was 20 °C. Before testing, nitrogen was purged 10 times. For each sample, the number of tests was 10 times, and the average of the test results was taken as the density of the sample.

#### 2.3.3. Equilibrium Swelling Measurement

Equilibrium swelling measurements were performed in the solvent toluene at 20 °C. The elastomer sample (about 6 × 5 × 3 mm^3^, ~0.1 g) was immersed in toluene, and the sample was taken out at intervals, the residual solvent on the surface was swept and weighed, and the sample was put back into the toluene solvent. The above-mentioned steps were repeated until the mass difference between two consecutive steps was less than 0.001 g. The equilibrium volume-swelling ratio ($q_{v}$) of the elastomer was calculated using Equation (1) [31], where $w_{0}$ is the initial mass of the sample, w is the mass after swelling, $\rho_{1}$ is the solvent density, and $\rho_{2}$ is the elastomer density.

(1) $$q_{v}=1+\left(\frac{w}{w_{0}}-1\right)\frac{\rho_{2}}{\rho_{1}}$$

#### 2.3.4. DSC Test

Elastomer samples were tested by using F204 differential scanning calorimeter (DSC, Netzsch). The instrument was temperature-corrected using indium standard (T_m_ = 156.6 °C). All tests were conducted under dry nitrogen atmosphere. Samples of 5–10 mg were first heated to 100 °C at a heating rate of 30 °C min^−1^, then held for 5 min to eliminate the thermal history. Then the samples were cooled to −50 °C at a cooling rate of 5 °C min^−1^ using liquid nitrogen. After that, the samples were heated again to 100 °C at a heating rate of 10 °C min^−1^, and the data of the secondary heating were recorded.

#### 2.3.5. Wide-Angle X-ray Diffraction Test

Wide-angle X-ray diffraction (WAXD) was performed on elastomer samples by using a MiniFlex 600 X-ray diffractometer (Gigaku) with Ni-filtered Cu Kα radiation (40 kV, 40 mA). The test was conducted at 20 °C with a scanning speed of 2° min^−1^, and the 2θ angle ranged from 5° to 40°.

#### 2.3.6. Mechanical Properties Test

Elastomer samples were tested by using a CMT4104 tensile tester (MTS). Elastomer samples were cut into dumbbell-shaped specimens (central portion 4 mm × 3 mm, gauge length 15 mm). The test temperature was 20 °C and the tensile rate was 20 mm min^−1^. The stress–strain curves were recorded with a minimum of three valid values for each group sample.

## 3. Result and Discussion

### 3.1. FT-IR of Elastomers with Different R Values

**Figure 2:**
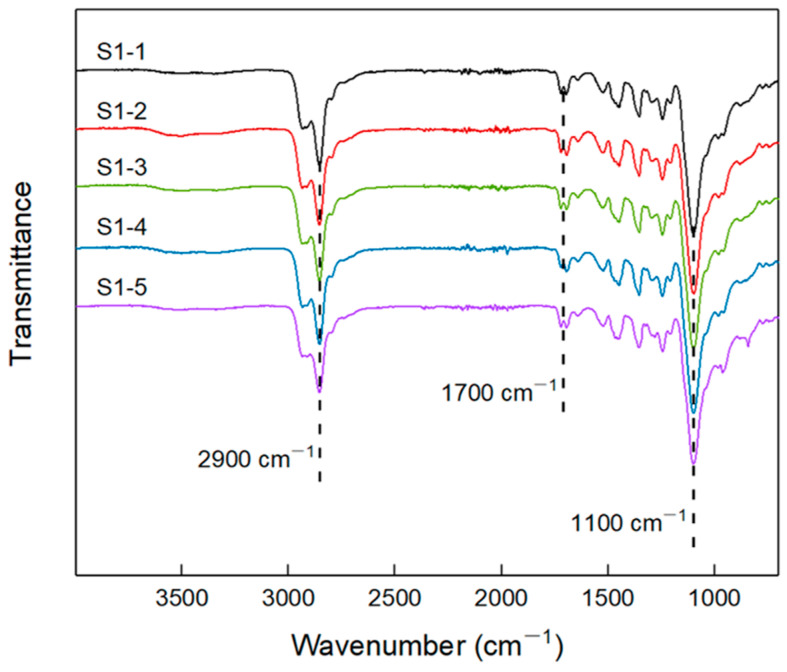
FTIR spectra of elastomers S1-1~S1-5.

The infrared spectra of elastomers S1-1~S1-5, which were prepared according to Table 1, are shown in Figure 2. It can be seen that there existed some typical group absorption peaks, among which: ~2900 cm^−1^ absorption peak corresponds to the C-H stretching vibration of -CH_2_- on the polyether chain of P(E-co-T) and PEG, and the absorption peak at ~1100 cm^−1^ is the stretching vibration of the ether bonds. All infrared spectra of elastomers S1-1~1–5 had no obvious absorption peaks at ~2260 cm^−1^, indicating that the isocyanate curing agent had completely reacted with the terminal hydroxyl of the blended polyether P(E-co-T)/PEG, having formed the carbamate structure. As a result, all infrared absorption spectra of elastomer S1-1~1–5 gave a characteristic peak at ~1700 cm^−1^ that was attributed to the formed carbamate group. 

### 3.2. Chemical Crosslinking Networks of Elastomers with Different R Values

**Figure 3:**
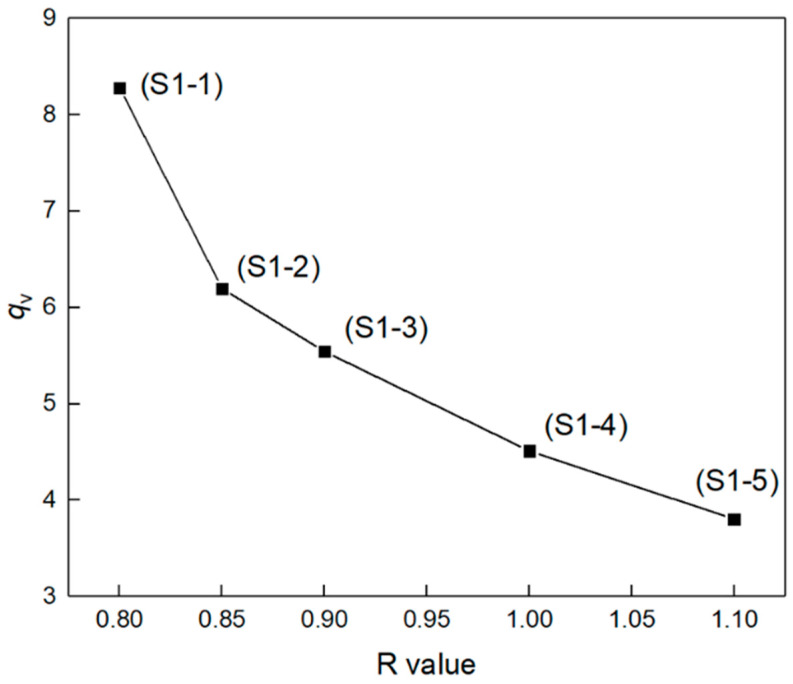
Equilibrium volume-swelling ratios of elastomers with different R values.

The chemical crosslinking network is one of the most important factors affecting elastomer mechanical properties. Elastomers S1-1~S1-5 were tested for swelling behaviors, and the relationship of equilibrium volume-swelling ratio (*q*_v_) dependent on R value is shown in Figure 3. Obviously, the equilibrium volume–swelling ratio of elastomers S1-1~S1-5 decreased monotonically with the increasing R value. Based on the Flory–Huggins theory [32], the apparent average molecular weight (*M*_c_) of the cross-linked elastomer network chain and the network chain density (*N*_0_) can be estimated using Equations (2)–(6) [28,31], where *ρ* is the density of the elastomer, g cm^−3^; V is the molar volume of the solvent toluene, 106.4 mL mol^−1^; $v_{2m}$ is the volume fraction of the elastomer; $\chi_{1}$ is the Flory–Huggins interaction parameter between elastomer and solvent, which was obtained using the Bristow–Watson equation (Equation (4)) [33]; the solvent toluene solubility parameter (*δ*_s_) is 18.241 (J cm^−3^)^1/2^ [30]; the PEG solubility parameter (*δ*_PEG_) is 18.473 (J cm^−3^)^1/2^ [34]; the P(E-co-T) solubility parameter (*δ*_P(E-co-T)_) is 18.357 (J cm^−3^)^1/2^; the solubility parameter of P(E-co-T)/PEG blended polyether elastomer (*δ*_P_) is obtained by weighting PEG and P(E-co-T) solubility parameters according to Equation (5), where *p* is the mass fraction of PEG in P(E-co-T)/PEG blended polyether.

(2) $$M_{c}=-V\rho \left(v_{2m}^{1/3}-\frac{v_{2m}}{2}\right)/\left[\ln\left(1-v_{2m}\right)+v_{2m}+\chi_{1}v_{2m}^{2}\right]$$

(3) $$v_{2m}=\frac{1}{q_{v}}$$

(4) $$\chi_{1}=0.34+\frac{V}{RT}{\left(\delta_{p}-\delta_{s}\right)}^{2}$$

(5) $$\delta_{p}=p\times \delta_{PEG}+\left(1-p\right)\times \delta_{PET}$$

(6) $$N_{0}=\frac{\rho }{M_{c}}$$

The network structure parameters of elastomer S1-1~S1-5 are listed in Table 3. With the increase in R value, the apparent average molecular weight $M_{c}$ of the elastomer S1-1~S1-5 decreased monotonically from 16416 g mol^−1^ to 3086 g mol^−1^, and the network chain density *N*_0_ increased monotonically from 0.0645 mmol cm^−3^ to 0.3437 mmol cm^−3^. This was because, under the condition of higher R value, the terminal hydroxyl groups of the blended polyether and the curing agent isocyanate had completely reacted; meanwhile, the excessive isocyanate group could further react with the resultant carbamate group to form a urea group. The elastomer network formed more chemical cross-linking points and the network chains presented lower average molecular weight *M*_c_ and higher network chain density *N*_0_. At lower R value, the terminal hydroxyl group of blended polyether could not react completely with the isocyanate curing agent, so that there existed a lot of suspended chains within the elastomer network. Consequently, the elastomer exhibited a higher apparent average molecular weight $M_{c}$ and a lower network chain density *N*_0_ [12].

### 3.3. Aggregation of Elastomers with Different R Values

**Figure 4:**
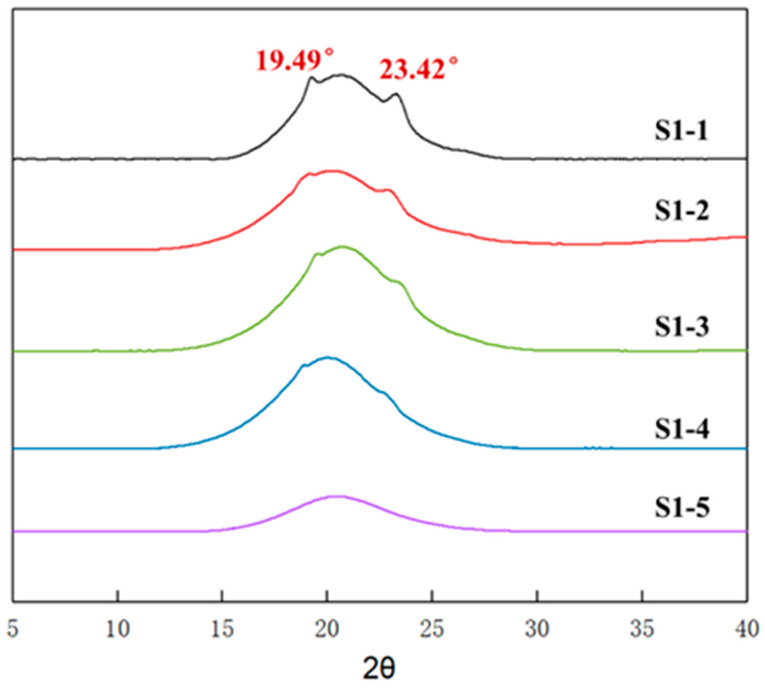
WAXD spectra of elastomers with different R values.

In addition to the chemical crosslinking, a physical crosslinking of the elastomers is also one of the important factors affecting the macro-mechanical properties. Figure 4 shows the wide-angle X-ray diffraction spectra of elastomers S1-1~S1-5 at room temperature. It can be seen that elastomers S1-1~S1-5 showed different X-ray diffraction characteristics. Elastomers S1-5 just showed a halo peak without sharp diffraction spikes. In contrast, the diffraction spectra of elastomers S1-1~S1-4 showed obvious diffraction spikes at 2θ angles 19.4° and 23.4°; moreover, the intensity of diffraction spikes gradually decreased with the increasing curing parameter R value. Given that the characteristic diffraction spikes of polytetrahydrofuran crystals appeared at 2θ angle 19.8°~20.0° and 24.1°~24.6° [35], while PEG crystals appeared at 2θ angle 19.2°~19.5° and 23.4°~23.6° [36], at room temperature, the diffraction spikes presented in elastomers S1-1~S1-4 should originate from the PEG crystalline structure.

**Figure 5:**
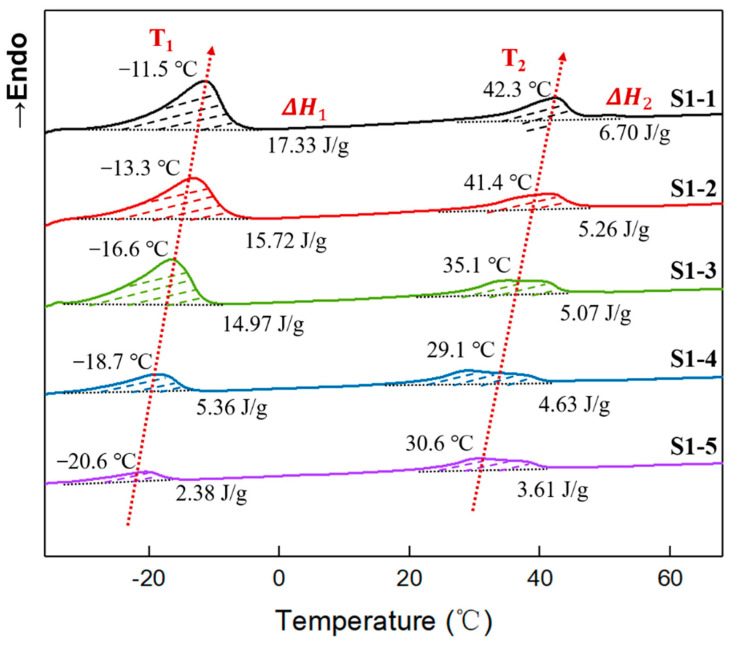
DSC curves of elastomers with different R values.

In addition, the thermal scans of elastomers S1-1~S1-5 were performed using DSC, and the secondary heating DSC curves are shown in Figure 5. It can be seen that two obvious endothermic peaks appeared during the secondary heating process for all samples. Given that the crystalline melting point of tetrahydrofuran micro-blocks occurred in the low-temperature region [9], while the crystalline melting point of polyethylene glycol occurred in the high temperature region [37], combined with the X-ray diffraction spectra of elastomer S1-1~S1-5 at room temperature (Figure 4), it can be inferred that, in Figure 5 the endothermic peak in the low-temperature region originated from the crystalline melting of the tetrahydrofuran micro-block on the P(E-co-T) chains [28], while the endothermic peak in the high-temperature region originated from the crystalline melting of polyethylene glycol.

Comparing the crystalline melt peaks in the high-temperature region, it was also found that the endothermic melting peaks of elastomers S1-3, S1-4, and S1-5, which were prepared by using higher curing parameter R values, was characteristic of a significantly wide distribution. Considering the high R value causing high network cross-linked density, the movement of PEG segments within the elastomers was limited, making them difficult to completely form thermodynamically stable aggregated structures. In the elastomer matrices, both PEG microcrystals and PEG structural intact crystal grains coexisted [37,38,39,40]. During heating, the PEG microcrystals first melted, followed by the structural intact crystal grains at higher temperatures, so the elastomers gave a wider distribution of endothermic peaks in the high-temperature region. As the R value decreased, the restriction of chain segment movement weakened, and PEG segment mobility increased, forming more thermodynamically stable and structurally intact crystal grains. Correspondingly, the PEG crystalline melting peak became narrower and shifted toward higher temperatures. In contrast, the microcrystalline structure formed by tetrahydrofuran micro-blocks exhibited only a single melting peak at low temperature. This indicates that, at room temperature, elastomers S1-1~S1-5 were all semi-crystalline aggregations caused by PEG crystallization.

### 3.4. Crystallinity of Elastomers with Different R Values

To further reveal the effect of crystallization physical crosslinking on the mechanical properties of elastomers S1-1~S1-5, the crystallinities have been quantitatively analyzed. As can be seen from Figure 5, the crystallization enthalpies of tetrahydrofuran micro-blocks and PEG gradually decreased with the increasing R value. The crystallization enthalpy of tetrahydrofuran micro-blocks decreased from 17.33 J g^−1^ for elastomer S 1-1 at R value of 0.80 to 2.38 J g^−1^ for elastomer S1-5 at R value of 1.10, while the crystallization enthalpy of PEG decreased from 6.70 J g^−1^ to 3.61 J g^−1^. Based on that polytetrahydrofuran crystallization enthalpy ${\Delta H}_{100\%}$ is 172.0 J g^−1^ [41], PEG crystallization enthalpy ${\Delta H}_{100\%}$ 156.98 J g^−1^ [38], for P(E-co-T)/PEG blended polyether elastomers, the crystallinities of tetrahydrofuran micro-blocks and PEG can be estimated using Equation (7), where ${\Delta H}_{m}\;$ is the melting enthalpy per unit mass of the elastomer.

(7) $$X_{c}=\frac{{\Delta H}_{m}}{{\Delta H}_{100\%}}\times 100\%$$

The crystallinities of elastomers S1-1~S1-5 are listed in Table 4. It can be seen that, within the P(E-co-T)/PEG elastomer, tetrahydrofuran micro-block crystallinity decreased from 10.08% for elastomer S1-1 at R value 0.80 to 1.38% for elastomer S1-5 at R value of 1.10, and PEG crystallinity decreased from 4.27% to 2.30%. The crystallinities of elastomers S1-1~S1-5 decreased with the increase of the curing parameter R value. As is consistent with XRD analysis. Considering that the crystalline melting peak of tetrahydrofuran micro-block is far lower than room temperature, while the melting peak of PEG is higher than room temperature, it can be inferred that PEG crystallinity, which dominates elastomer physical crosslinking degree at room temperature, would have a significant influence on the mechanical properties of P(E-co-T)/PEG blended polyether elastomer at room temperature.

### 3.5. Mechanical Properties of Elastomers with Different R Values

**Figure 6:**
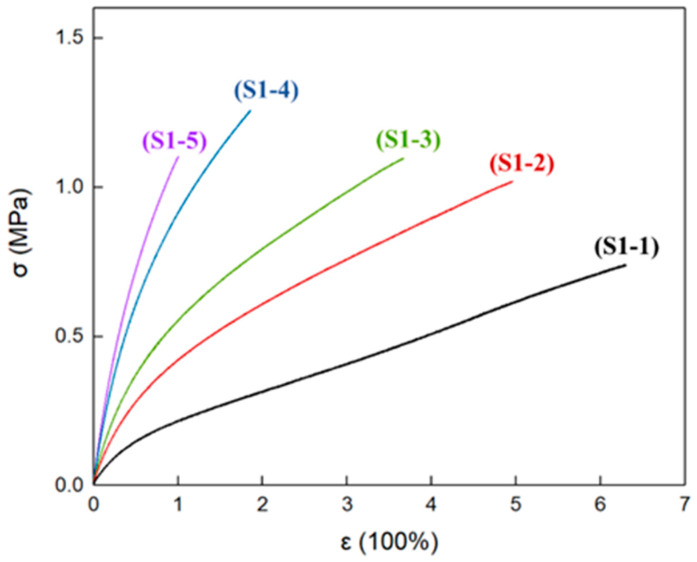
Stress–strain curves of elastomers with different R values at room temperature.

At room temperature, the typical stress–strain curves of P(E-co-T)/PEG blended polyether elastomer S1-1~S1-5 are shown in Figure 6. The tensile moduli (*E*) of elastomer S1-1~S1-5 gradually increase with the increase in R value, indicating that, under the condition that P(E-co-T) and PEG content remain unchanged, the elastomer tensile modulus mainly depended on the elastomer curing parameters. Higher curing parameters and higher chemical crosslinking density are favorable to the elastomer tensile modulus. As to the elongation at break (***ε*_b_**) and the tensile strength at break (***σ*_b_**) of the elastomers, the ***ε*_b_** gradually decreased as the curing parameter increased, while the ***σ*_b_** first increased and then decreased.

Table 5 lists the mechanical property results of elastomers S1-1~S1-5. Clearly, elastomers S1-2 and S1-3 had similar tensile strength at break as elastomer S1-5, but had higher elongation. Combined with the crystallinity of the elastomers at room temperature (Figure 4 and Table 4), it can be inferred that, within elastomer S1-2 and S1-3 matrices, the physical crosslinking structure formed by PEG crystallization compensated for the adverse effect of the lower curing parameter R value on the mechanical properties of the elastomers, making elastomers S1-2 and S1-3 simultaneously give excellent elongation at break and tensile strength at break.

Mechanical properties are a macroscopic manifestation of elastomer microstructure characteristics. To confirm the micro-aggregation thermal stability of elastomers S1-1~S1-5, the elastomers were heated and aged at 60 °C for 7 days, and then the mechanical properties were tested at room temperature. Table 6 lists the mechanical property results of the elastomers after aging. Comparing with Table 5, there was no obvious difference in mechanical properties of the elastomers before and after aging. This suggests that the micro-aggregation of elastomers S1-1~S1-5 had not noticeably changed after long-term high-temperature aging, and the micro-aggregation of polyurethane crosslinked P(E-co-T)/PEG elastomer had good thermal stability. 

### 3.6. Aggregation of Elastomers with Different PEG Contents

**Figure 7:**
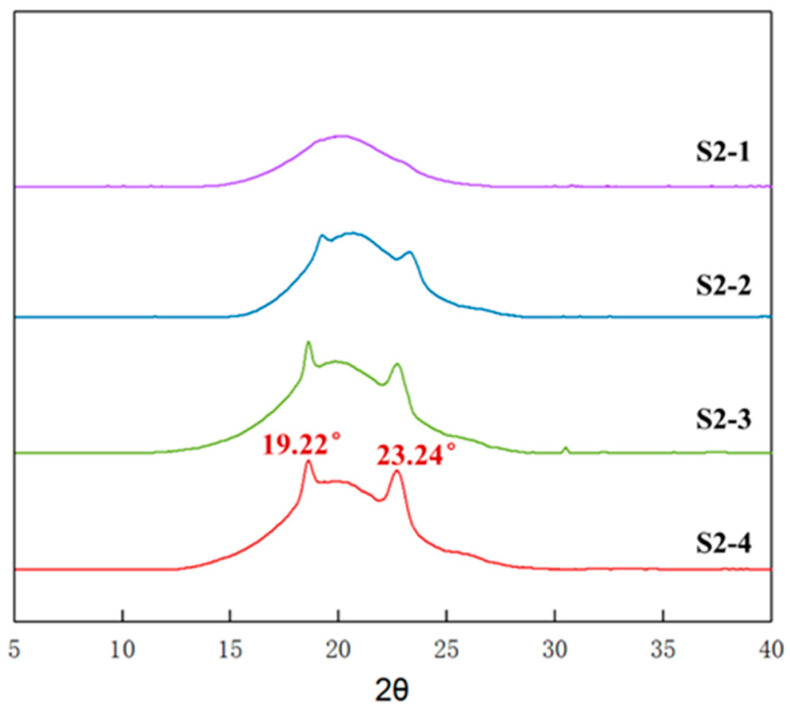
WAXD spectra of elastomers with different PEG contents.

To further explore the effect of PEG content on the mechanical properties of P(E-co-T)/PEG blended polyether elastomers, fixing the curing parameter at 0.9 and varying the PEG content according to Table 2, elastomers S2-1~S2-4 with different PEG contents were prepared and analyzed by using X-ray diffraction. Figure 7 shows the X-ray diffraction spectra of elastomer S2-1~S2-4 at room temperature. Obviously, there emerged noticeable diffraction spikes around 2θ angle 19.2° and 23.2°, and the intensity gradually increased as PEG content increased from 8% of S2-1 to 20% of S2-4. Referring to Figure 4, it can be inferred that the diffraction spikes originated from the crystalline structure of PEG segments within P(E-co-T)/PEG blended polyether elastomer. 

### 3.7. Crystallinity of Elastomers with Different PEG Contents

**Figure 8:**
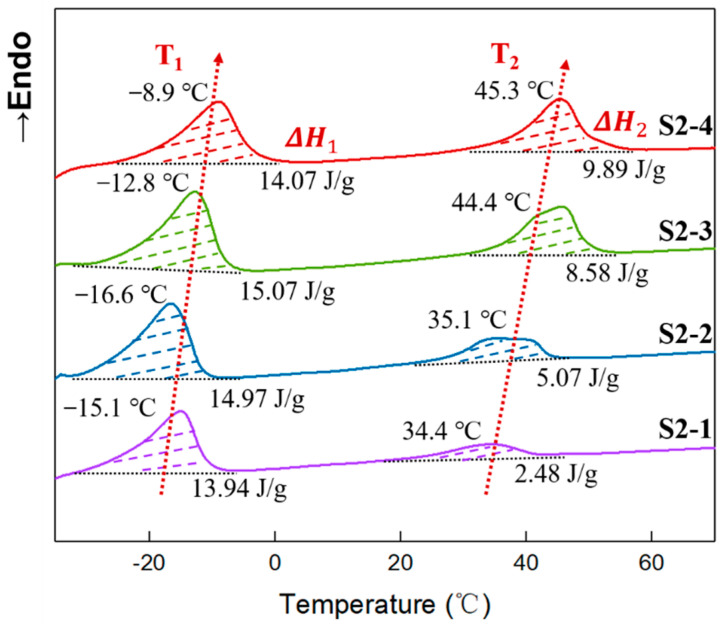
DSC curves of elastomers with different PEG contents.

The secondary heating DSC curves of elastomers S2-1~S2-4 are shown in Figure 8. All elastomer samples presented two obvious endothermic peaks. Similarly, the endothermic peak below room temperature is attributed to the crystalline melting of the tetrahydrofuran micro-block on the P(E-co-T) chain, while the one above room temperature was the crystalline melting of PEG. As in Figure 5, both crystalline melting peak temperatures showed an overall trend toward higher temperatures with increasing PEG content. This indicates that more structural intact crystal grains were formed within P(E-co-T)/PEG blended polyether elastomer as the PEG content increased, leading to a shift of the crystalline melting peak toward higher temperatures. Additionally, within P(E-co-T)/PEG blended polyether elastomer matrices, increasing PEG content contributed to the integrity of the crystalline structure.

Based on the melting enthalpy of Figure 8, the crystallinity of P(E-co-T)/PEG elastomers S2-1 to S2-4, which were calculated using Equation (7), are shown in Table 7. It can be seen that the crystallinity of tetrahydrofuran micro-block was in the range of 8.10–8.76% with the increase in PEG content; in contrast, the crystallinity of PEG rose from 1.58% of elastomer S2-1 to 6.30% of elastomer S2-4, having increased by ~300%. This indicates that, within P(E-co-T)/PEG blended polyether elastomers, fixing curing parameter R value, increasing PEG content could significantly enhance the physical crosslinking degree of the elastomer at room temperature.

### 3.8. Mechanical Properties of Elastomers with Different PEG Contents

**Figure 9:**
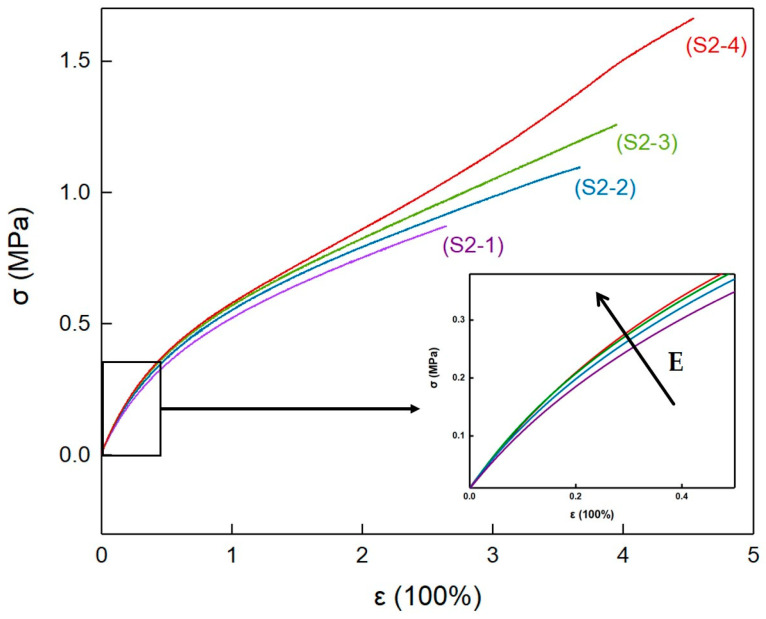
Stress–strain curves of elastomers with different PEG contents.

The mechanical properties of elastomers S2-1~S2-4 were tested at room temperature, and the typical stress–strain curves are shown in Figure 9. At the beginning of the straining, the stress–strain curves of elastomers S2-1~S2-4 all exhibited a typical tensile behavior of amorphous polymer elastomers, and the tensile modulus gradually decreased with increasing strain. In addition, combined with Table 7, it can be inferred that high crystallinity increased the physical crosslinking density of the elastomers. This made the tensile modulus of elastomers S2-1~S2-4 gradually increase with the increase in PEG content. 

At the late strain stage, the tensile modulus of elastomer S2-4 showed a significant upturn trend with increasing strain. This indicates that, within P(E-co-T)/PEG blended polyether elastomers, when PEG content increased to 20%, the elastomer network chains orientated and further crystallized during the strain process, increasing the physical crosslinking density and causing the modulus increase. At the same time, the oriented crystallization allowed the network chains to further relax, making elastomers S2-4 have a higher strain and tensile strength at break.

Table 8 lists the mechanical property results of elastomers S2-1~S2-4. The tensile modulus increased from 0.78 MPa for elastomer S2-1 to 0.93 MPa for elastomer S2-4, and the elongation at break increased from 237% for elastomer S2-1 to 443% for elastomer S2-4, and the tensile strength at break increased from 0.80 MPa for elastomer S2-1 to 1.49 MPa for elastomer S2-4. The initial tensile modulus, elongation at break, and tensile strength all gradually increased with the increase in PEG content. The introduction of crystallizable PEG to polyurethane cross-linked P(E-co-T) elastomers could change the micro-aggregation structure of the elastomers at room-temperature, and significantly improve the mechanical properties.

## 4. Conclusions

The polyurethane cross-linked P(E-co-T)/PEG blended polyether elastomers with different curing parameter R values and different PEG contents were prepared by using N100 as the curing agent. When PEG content was 12%, polyurethane cross-linked P(E-co-T)/PEG blended polyether elastomer presented a semi-crystalline structure at room temperature, and the crystallinity gradually increased with the decrease in curing parameter R value. At R value 0.9, the elastomer exhibited both good tensile strength and elongation at break. Fixing the curing parameter R value at 0.9, all the elastomer tensile strength, tensile modulus, and elongation at break gradually increased with increasing PEG content. Introducing crystallizable PEG into polyurethane cross-linked P(E-co-T) elastomers could effectively adjust elastomer micro-aggregation at room temperature and improve the elastomer mechanical properties. 

Apart from utilizing prepolymer molecular weight, curing agent type, and curing parameters to regulate the mechanical properties of thermoset elastomers, introducing crystallizable prepolymers and using the crystallization physical crosslinking are important ways to regulate the mechanical properties of amorphous thermoset elastomers.

## Figures and Tables

**Table 1:** Formulation composition of elastomers with different R values.

Samples	R Value	P(E-co-T) (g)	PEG (g)	N100 (g)	T_12_ (g)
S1-1	0.80	17.6	2.4	1.409	0.001
S1-2	0.85	17.6	2.4	1.494	0.001
S1-3	0.90	17.6	2.4	1.582	0.001
S1-4	1.00	17.6	2.4	1.757	0.001
S1-5	1.10	17.6	2.4	1.933	0.001

**Table 2:** Formulation composition of elastomers with different PEG contents.

Samples	PEG (wt%)	P(E-co-T) (g)	PEG (g)	N100 (g)	T_12_ (g)
S2-1	8	18.4	1.6	1.577	0.001
S2-2	12	17.6	2.4	1.582	0.001
S2-3	16	16.8	3.2	1.586	0.001
S2-4	20	16.0	4.0	1.590	0.001

**Table 3:** Network structure parameters of elastomers with different R values.

Samples	ρ (g cm^−3^)	$\delta_{p}{\left(J\cdot cm^{-3}\right)}^{\frac{1}{2}}$	$\chi_{1}$	$q_{v}$	$v_{2m}$	$M_{c}$ (g mol^−1^)	$N_{0}$ (mmol cm^−3^)
S1-1	1.0594	18.371	0.341	8.2806	0.1208	16,416	0.0645
S1-2	1.0609	18.371	0.341	6.1976	0.1614	9087	0.1168
S1-3	1.0623	18.371	0.341	5.5454	0.1803	7178	0.1478
S1-4	1.0577	18.371	0.341	4.5106	0.2217	4549	0.2325
S1-5	1.0604	18.371	0.341	3.8050	0.2628	3086	0.3437

**Table 4:** Melting enthalpy and crystallinity of elastomers with different R value.

Samples	T_1_ (°C)	$\Delta H_{1}$ (J/g)	$X_{c}$ (%)	T_2_ (°C)	$\Delta H_{2}$ (J/g)	$X_{c}$ (%)
S1-1	−11.5	17.33	10.08	42.3	6.70	4.27
S1-2	−13.3	15.72	9.14	41.4	5.26	3.35
S1-3	−16.6	14.97	8.70	35.1	5.07	3.23
S1-4	−18.7	5.36	3.12	29.1	4.63	2.95
S1-5	−20.6	2.38	1.38	30.6	3.61	2.30

**Table 5:** Mechanical properties of elastomers with different R values.

Samples	R Value	*ε*_b_ (%)	*σ*_b_ (MPa)	*E* (MPa)
S1-1	0.80	630	0.738	0.356
		617	0.738	0.348
		588	0.727	0.362
		612 ± 5	0.73 ± 0.01	0.36 ± 0.01
S1-2	0.85	408	0.972	0.663
		448	0.972	0.675
		496	1.018	0.614
		451 ± 7	0.99 ± 0.03	0.65 ± 0.03
S1-3	0.90	312	0.980	0.809
		367	1.096	0.863
		370	1.111	0.880
		349 ± 6	1.06 ± 0.07	0.85 ± 0.03
S1-4	1.00	169	1.173	1.411
		177	1.185	1.376
		185	1.258	1.453
		177 ± 3	1.20 ± 0.04	1.41 ± 0.03
S1-5	1.10	81	0.980	1.700
		88	1.018	1.616
		100	1.101	1.717
		90 ± 3	1.03 ± 0.06	1.68 ± 0.05

**Table 6:** Mechanical properties of aged elastomers with different R values.

Sample	R Value	*ε*_b_ (%)	*σ*_b_ (MPa)	*E* (MPa)
S1-1	0.80	635	0.730	0.350
		622	0.731	0.346
		614	0.721	0.339
		624 ± 4	0.73 ± 0.01	0.35 ± 0.01
S1-2	0.85	459	0.988	0.664
		497	0.963	0.657
		489	0.964	0.652
		482 ± 5	0.97 ± 0.02	0.66 ± 0.01
S1-3	0.90	331	0.999	0.787
		359	0.981	0.851
		376	1.055	0.780
		355 ± 5	1.01 ± 0.03	0.81 ± 0.04
S1-4	1.00	180	1.214	1.411
		192	1.198	1.379
		176	1.186	1.396
		183 ± 3	1.20 ± 0.01	1.40 ± 0.02
S1-5	1.10	114	1.034	1.680
		97	0.991	1.608
		86	1.011	1.721
		99 ± 3	1.01 ± 0.02	1.66 ± 0.05

**Table 7:** Melting enthalpy and crystallinity of elastomers with different PEG contents.

Samples	T_1_ (°C)	$\Delta H_{1}$ (J/g)	$X_{c}$ (%)	T_2_ (°C)	$\Delta H_{2}$ (J/g)	$X_{c}$ (%)
S2-1	−15.1	13.94	8.10	34.4	2.48	1.58
S2-2	−16.6	14.97	8.70	35.1	5.07	3.23
S2-3	−12.8	15.07	8.76	45.7	8.58	5.47
S2-4	−8.9	14.07	8.18	45.3	9.89	6.30

**Table 8:** Mechanical properties of elastomers with different PEG contents.

Samples	ε (%)	σ (MPa)	E (MPa)
S2-1	264	0.872	0.820
	224	0.771	0.777
	223	0.746	0.735
	237 ± 5	0.80 ± 0.07	0.78 ± 0.04
S2-2	312	0.980	0.809
	367	1.096	0.863
	370	1.111	0.880
	331 ± 7	0.84 ± 0.02	1.03 ± 0.03
S2-3	339	1.170	0.962
	349	1.157	0.931
	394	1.259	0.881
	361 ± 5	1.20 ± 0.06	0.92 ± 0.04
S2-4	427	1.428	0.918
	449	1.467	0.921
	454	1.563	0.943
	443 ± 4	1.49 ± 0.06	0.93 ± 0.01

## Data Availability

The data presented in this study are available on request from the corresponding author.

## References

[1] Engels H.W., Pirkl H.G., Albers R., Albach R.W., Krause J., Hoffmann A., Casselmann H., Dormish J. (2013). Polyurethanes: Versatile materials and sustainable problem solvers for today’s challenges. J. Ger. Chem. Soc..

[2] Berezkin Y., Urick M. (2013). Modern Polyurethanes: Overview of Structure Property Relationship. Polymers for Personal Care and Cosmetics.

[3] Tian S. (2020). Recent Advances in Functional Polyurethane and Its Application in Leather Manufacture: A Review. Polymers.

[4] Chen H.M., Li X.P., Chen J., He X.D., Huang W.M., Zhu K., Yu W.H., Ni H.L., Zhao K.Q., Hu P. (2021). Unified method to prepare thermoplastic/thermoset soft polyurethanes reshape-able around room temperature on-demand. J. Polym. Res..

[5] Meiorin C., Calvo-Correas T., Mosiewicki M.A., Aranguren M.I., Corcuera M.A., Eceiza A. (2019). Comparative effects of two different crosslinkers on the properties of vegetable oil-based polyurethanes. J. Appl. Polym. Sci..

[6] Li B., Zhao Y., Liu G., Li X., Luo Y. (2016). Mechanical properties and thermal decomposition of PBAMO/GAP random block ETPE. J. Therm. Anal. Calorim..

[7] Zhang C., Li J., Luo Y. (2012). Synthesis and Characterization of 3,3′-Bisazidomethyl Oxetane-3-Azidomethyl-3′-Methyl Oxetane Alternative Block Energetic Thermoplastic Elastomer. Propellants Explos. Pyrotech..

[8] Eroglu M.S., Guven O. (1998). Characterization of network structure of poly(glycidyl azide) elastomers by swelling, solubility and mechanical measurements. Polymer.

[9] Li Y., Li J., Ma S., Luo Y. (2017). Compatibility, mechanical and thermal properties of GAP/P(EO-co-THF) blends obtained upon a urethane-curing reaction. Polym. Bull..

[10] Chen K., Yuan S., Wen X., Sang C., Luo Y. (2021). Effect of Mixed Isocyanate Curing Agents on the Performance of In Situ-Prepared HTPE Binder Applied in Propellant. Propellants Explos. Pyrotech..

[11] Jutrzenka Trzebiatowska P., Santamaria Echart A., Calvo Correas T., Eceiza A., Datta J. (2018). The changes of crosslink density of polyurethanes synthesised with using recycled component. Chemical structure and mechanical properties investigations. Prog. Org. Coat..

[12] Zhai J.X., Pang A.M., Ding T.F., Liu R.T., Guo X.Y., Song T.L. (2022). Effect of crosslinking point structures on properties of polyurethane end-crosslinked PBT elastomers. Iran. Polym. J..

[13] Zheng Q., Wang G., Du J., Li J., Li J., Tang Q., Fan X. (2020). Investigation of Hydroxyl-Terminated Polyether Cured with Different Isocyanates: Curing Process and Mechanical Property. Propellants Explos. Pyrotech..

[14] Liu Q., Liu Y., Zheng H., Li C., Zhang Y., Zhang Q. (2020). Design and development of self-repairable and recyclable crosslinked poly(thiourethane-urethane) via enhanced aliphatic disulfide chemistry. J. Polym. Sci..

[15] Kojio K., Fukumaru T., Furukawa M. (2004). Highly Softened Polyurethane Elastomer Synthesized with Novel 1,2-Bis(isocyanate)ethoxyethane. Macromolecules.

[16] Geng Z., Pang A., Ding T., Guo X., Yang R., Luo Y., Zhai J. (2022). Overlooked Impact of Interchain H-Bonding between Cross-Links on the Mechanical Properties of Thermoset Polyurethane Elastomers. Macromolecules.

[17] de Keer L., Kilic K.I., Van Steenberge P.H.M., Daelemans L., Kodura D., Frisch H., De Clerck K., Reyniers M.F., Barner-Kowollik C., Dauskardt R.H. (2021). Computational prediction of the molecular configuration of three-dimensional network polymers. Nat. Mater..

[18] de Keer L., van Steenberge P.H.M., Reyniers M.F., D’Hooge D.R. (2021). Going Beyond the Carothers, Flory and Stockmayer Equation by Including Cyclization Reactions and Mobility Constraints. Polymers.

[19] Nofar M., Mohammadi M., Carreau P.J. (2020). Effect of TPU hard segment content on the rheological and mechanical properties of PLA/TPU blends. J. Appl. Polym. Sci..

[20] Shen Z., Zheng L., Li C., Liu G., Xiao Y., Wu S., Liu J., Zhang B. (2019). A comparison of non-isocyanate and HDI-based poly(ether urethane): Structure and properties. Polymer.

[21] Schimpf V., Max J.B., Stolz B., Heck B., Mülhaupt R. (2018). Semicrystalline Non-Isocyanate Polyhydroxyurethanes as Thermoplastics and Thermoplastic Elastomers and Their Use in 3D Printing by Fused Filament Fabrication. Macromolecules.

[22] Eceiza A., Martin M.D., de la Caba K., Kortaberria G., Gabilondo N., Corcuera M.A., Mondragon I. (2008). Thermoplastic polyurethane elastomers based on polycarbonate diols with different soft segment molecular weight and chemical structure: Mechanical and thermal properties. Polym. Eng. Sci..

[23] Anokhin D.V., Gorbunova M.A., Abukaev A.F., Ivanov D.A. (2021). Multiblock Thermoplastic Polyurethanes: In Situ Studies of Structural and Morphological Evolution under Strain. Materials.

[24] Gorbunova M.A., Komov E.V., Grunin L.Y., Ivanova M.S., Abukaev A.F., Imamutdinova A.M., Ivanov D.A., Anokhin D.V. (2022). The effect of separation of blocks on the crystallization kinetics and phase composition of poly(butylene adipate) in multi-block thermoplastic polyurethanes. Phys. Chem. Chem. Phys..

[25] Liu Y., Gao J., Wang Y., Zhou J., Cao L., He Z., Zhang Y., Tang C., Zhong L. (2019). Enhanced Temperature Stability of High Energy Density Ferroelectric Polymer Blends: The Spatial Confinement Effect. Macromol. Rapid Commun..

[26] Alvarado N., Alegría L., Sandoval C., Kortaberría G., Leiva A., Gargallo L., Radic D. (2014). Synthesis and Characterization of a Branched Poly(Methacrylamide): Thermal Stability and Molecular Simulation Studies of Their Blends With Vinylic Polymers. J. Macromol. Sci. Part A.

[27] Karim A., Liu D.W., Douglas J.F., Nakatani A.I., Amis E.J. (2000). Modification of the phase stability of polymer blends by fillers. Polymer.

[28] Zou Y.C., Yang R.J., Zhai J.X. (2017). Polytriazole polyether elastomers with widely tunable mechanical properties: The role of network structure and crystallization behavior. J. Appl. Polym. Sci..

[29] Kambe Y. (1980). Thermal-Behavior of Poly(Ethylene Oxide) as Revealed by Differential Scanning Calorimetry. Polymer.

[30] van Krevelen D.W. (2009). Properties of Polymers: Their Correlation with Chemical Structure; Their Numerical Estimation and Prediction from Additive Group Contributions.

[31] Qu Z., Zhai J., Yang R. (2014). Comparison between properties of polyether polytriazole elastomers and polyether polyurethane elastomers. Polym. Adv. Technol..

[32] Flory P.J., Rehner J. (1943). Statistical Mechanics of Cross-Linked Polymer Networks II. Swelling. J. Chem. Phys..

[33] Bristow G.M., Watson W.F. (1958). Cohesive Energy Densities of Polymers Part 1—Cohesive Energy Densities of Rubbers by Swelling Measurements. Trans. Faraday Soc..

[34] Ozdemir C., Guner A. (2007). Solubility profiles of poly(ethylene glycol)/solvent systems, I: Qualitative comparison of solubility parameter approaches. Eur. Polym. J..

[35] Zhang L., Shi H., Li W., Han X., Zhang X. (2014). Thermal performance and crystallization behavior of poly(ethylene glycol) hexadecyl ether in confined environment. Polym. Int..

[36] Rachmawati R., Woortman A.J., Loos K. (2013). Facile preparation method for inclusion complexes between amylose and polytetrahydrofurans. Biomacromolecules.

[37] Yang J., Zhao T., Cui J., Liu L., Zhou Y., Li G., Zhou E., Chen X. (2006). Nonisothermal crystallization behavior of the poly(ethylene glycol) block in poly(L-lactide)–poly(ethylene glycol) diblock copolymers: Effect of the poly(L-lactide) block length. J. Polym. Sci. Part B Polym. Phys..

[38] Buckley C.P., Kovacs A.J. (1976). Melting behavior of low-molecular weight poly (ethylene-oxide) fractions. 2. folded chain crystals. Colloid Polym. Sci..

[39] Liu M., Zhao Q., Wang Y., Zhang C., Mo Z., Cao S. (2003). Melting behaviors, isothermal and non-isothermal crystallization kinetics of nylon 1212. Polymer.

[40] Run M., Wu S., Zhang D., Wu G. (2005). Melting behaviors and isothermal crystallization kinetics of poly(ethylene terephthalate)/mesoporous molecular sieve composite. Polymer.

[41] Jenkins M.J., Cao Y., Kukureka S.N. (2006). The effect of molecular weight on the crystallization kinetics and equilibrium melting temperature of poly(tetramethylene ether glycol). Polym. Adv. Technol..

